# Role of Radiographic Fibrosis Extent in Identifying Immunomodulatory Treatment Response in Hypersensitivity Pneumonitis

**DOI:** 10.1016/j.chest.2025.12.007

**Published:** 2025-12-18

**Authors:** Caroline N. Muegge, Bohyung Min, Ali Mrad, Emily Ianazzo, Mark Hamblin, Sahil Pandya, Gregory Lee, Melissa Carroll, Kerri A. Johannson, Scott M. Matson

**Affiliations:** a Department of Internal Medicine, University of Kansas Medical Center, Kansas City, KS; b Department of Radiology, University of Kansas Medical Center, Kansas City, KS; c Division of Pulmonary, Critical Care and Sleep Medicine, University of Kansas School of Medicine, Kansas City, KS; d Division of Pulmonary and Critical Care, University of Wisconsin Health, Madison, WI; e Department of Medicine, University of Calgary, Calgary, AB, Canada

**Keywords:** fibrosis, hypersensitivity pneumonitis, immunomodulation, treatment response

## Abstract

**Background:**

Treatment selection in chronic hypersensitivity pneumonitis (HP) remains empiric because of a lack of randomized trial data. Although radiographic fibrosis extent often is used to inform treatment decisions, its usefulness as a theragnostic marker for immunomodulatory therapy is unknown.

**Research Question:**

Is visual fibrosis extent on high-resolution CT (HRCT) imaging associated with differential pulmonary function response to immunomodulation in chronic HP?

**Study Design and Methods:**

This retrospective cohort study included 108 patients with HP from 2 interstitial lung disease (ILD) referral centers who received ≥ 3 months of immunomodulatory therapy (prednisone, azathioprine, mycophenolate mofetil, and rituximab) and had undergone pulmonary function testing and HRCT imaging before and after treatment. Fibrosis extent was classified as ≥ 10% or < 10% based on masked radiologist reads. Linear spline mixed-effects models were used to estimate FVC and diffusion capacity of the lungs for carbon monoxide (Dlco) % predicted trajectory before and after immunomodulation, with patients serving as their own controls. The primary analysis evaluated whether fibrosis extent modified the association between immunomodulation and lung function trajectory.

**Results:**

Overall, immunomodulation was associated with a modest FVC improvement at 12 months (+2.63%; 95% CI, 0.72-4.54; *P* < .01). Among patients with < 10% fibrosis, immunomodulation was associated with improvements in both FVC (+5.83%; *P* < .01) and Dlco (+13.9%; *P* < .01). In contrast, no improvement in FVC (+0.81%; *P* = .42) or Dlco (–4.3%; *P* = .17) was observed in patients with ≥ 10% fibrosis at 12 months. Baseline demographics, smoking history, and antigen identification status were similar between fibrosis groups.

**Interpretation:**

The results of this study indicate that visual fibrosis extent is associated with differential pulmonary function response to immunomodulatory therapy in chronic HP. Patients with limited fibrosis demonstrated improved pulmonary function compared with those with greater radiographic fibrosis. These findings may support the use of fibrosis extent as a clinical tool for treatment stratification and highlight the need for prospective trials to validate radiographic markers in guiding HP management.

**Take-Home Points**

**Research Question:** Is visual fibrosis extent on high-resolution CT imaging associated with differential pulmonary function response to immunomodulatory therapy in chronic hypersensitivity pneumonitis (HP)?

**Results:** Patients with < 10% visual fibrosis demonstrated significant improvement in FVC and diffusion capacity of the lungs for carbon monoxide after immunomodulation, whereas those with ≥ 10% fibrosis showed no meaningful lung function benefit.

**Interpretation:** Visual fibrosis extent on HRCT imaging may help to identify patients with chronic HP most likely to benefit from immunomodulatory therapy and could serve as a practical, clinically actionable tool for treatment stratification.

Hypersensitivity pneumonitis (HP) is an interstitial lung disease (ILD) caused by exposure to inhaled allergens, triggering an immune-mediated inflammatory response with subsequent lung injury and, in some patients, resulting lung fibrosis.^1^ Diagnosis and management of HP remains challenging because of heterogenous clinical and radiographic presentations and lack of randomized control trials to guide treatment.^2^ The most recent clinical guidelines propose the dichotomization of HP into 2 subtypes, nonfibrotic (purely inflammatory) and fibrotic (mixed inflammatory and fibrotic or purely fibrotic), based largely on high-resolution CT (HRCT) imaging and histopathologic features.^3^^,^^4^ This distinction carries clinical relevance because retrospective studies have shown that radiographic phenotype and extent of radiologic fibrosis correlates with prognosis.^5^, ^6^, ^7^, ^8^

It is widely accepted that the first-line therapy in HP is antigen avoidance^1^; however, meaningful decisions still are made regarding treatment choice when patients show ongoing progression. On one hand, observational studies have demonstrated that immunomodulatory therapy in patients with HP stabilized diffusion capacity of the lungs for carbon monoxide (Dlco) or FVC.^9^, ^10^, ^11^ On the other hand, nintedanib has demonstrated a reduction in FVC decline when compared with placebo for patients with progressive, fibrotic ILD (including patients with HP), although the main trial was not powered to evaluate individual subgroups.^12^^,^^13^

In the absence of high-quality randomized data to guide this decision between 2 treatment strategies, many experts and guidelines have recommended adopting radiologic patterns such as the usual interstitial pneumonia pattern or visual extents of fibrosis to categorize patients into those most likely to respond positively to immunomodulation vs those most likely to benefit from an antifibrotic approach.^1^^,^^14^^,^^15^ However, as we have shown previously in autoimmune ILD, prognostic markers such as usual interstitial pneumonia or extent of fibrosis may not be associated with differential response to immunomodulation, and therefore may not be an appropriate theragnostic marker for these important treatment decisions in HP.^16^^,^^17^ This study aimed to address that gap by evaluating the association between FVC and Dlco response to immunosuppression and the visual extent of fibrosis on pretreatment CT scans in patients with HP.

## Study Design and Methods

### Study Participants

Patients were identified from 2 ILD referral centers: the University of Kansas Medical Center and the University of Calgary. Inclusion criteria required that participants: (1) carried a clinical diagnosis of HP assigned by an ILD pulmonologist; (2) had initiated immunomodulatory therapy for HP and received at least 3 months of treatment, as confirmed by medical record review; (3) had at least 1 set of baseline pulmonary function testing results available before initiation of therapy (within the 3 months before treatment initiation) and had undergone at least 1 follow-up pulmonary function testing within 12 months of starting therapy; and (4) underwent high-resolution CT (HRCT) imaging within 3 months before treatment initiation.

Antigen identification was determined pragmatically based on documentation in the clinical chart. If the treating ILD pulmonologist referred to a specific antigen exposure in follow-up notes, this was considered sufficient to count the antigen as identified. Immunomodulatory therapy was defined as treatment with azathioprine, mycophenolate mofetil, rituximab, prednisone (administered at ≥ 10 mg/d for at least 3 months), or any combination thereof. A total of 108 patients met all inclusion criteria across both sites. Clinical data collection included baseline demographics, treatment exposure, radiographic features, and serial pulmonary function data (FVC and Dlco with corresponding test dates) before and after initiation of immunomodulatory therapy. The study was approved by the University of Kansas institutional review board on December 21, 2023 (Identifier: STUDY00160431).

### Radiographic Analysis

Pretreatment HRCT scans were reviewed independently by 2 thoracic radiologists who were masked to the rest of the study design. Evaluation of fibrosis extent was used to categorize patients into 2 radiographic categories: visual fibrosis extent ≥ 10% and visual fibrosis extent < 10%, as has been carried out in ILD trials previously.^12^^,^^18^ Masked radiologists also determined ILD pattern based on published criteria.^19^ Discrepancies in classification between the 2 reading radiologists were resolved via consensus in a fashion as described previously.^16^^,^^20^

### Statistical Analysis

A linear mixed-effects spline model was used for the primary and secondary analyses of lung function trajectory (FVC or Dlco) trends before and after treatment initiation with immunomodulation. For a specific FVC (or Dlco) measurement, the time variable was calculated as the date of the FVC (or Dlco) minus the baseline FVC (or Dlco) date before treatment initiation converted into months. Therefore, time 0 in these models reflect the baseline pulmonary function testing measurement before one of the candidate immunomodulatory agents was started, and all negative times on these plots reflect lung function measured before treatment. A linear spline mixed model with random intercept including FVC % predicted (or Dlco % predicted) as dependent variable was fit with time 0 as the knot using all eligible patients. The mean response after 12 months of treatment was compared with the counterfactual value of FVC % predicted (or Dlco % predicted) had the pretreatment trend continued past time 0 to > 12 months for the primary analyses. Therefore, these comparisons are the actual after-treatment values of lung function over time with the extrapolated pretreatment trajectory, allowing these patients to serve as their own controls.

To examine if visual extent of fibrosis was associated with differential treatment response, we generated these linear spline models across the 108 patients in 2 prespecified groups: (1) patients with visual fibrosis score of ≥ 10% and (2) those with fibrosis score of < 10%. Multivariable spline models were adjusted for age, sex, current smoking status, treatment center, and the baseline value of the corresponding outcome variable (ie, baseline FVC % predicted for the FVC model and baseline Dlco % predicted for the Dlco model). Repeated measurements over time were accounted for by including a random intercept for patient identifier, allowing each patient to contribute multiple lung function measurements to the model while accounting for intraindividual correlation.

To assess formally whether posttreatment trajectories differed by fibrosis extent, we fit a single linear mixed-effects spline model with a knot at treatment (month 0), including fixed effects for time (pretreatment slope), time since treatment initiation (post) (posttreatment slope, defined as post = max(time, 0)), fibrosis extent (< 10% vs ≥ 10%), and their interaction terms (time × fibrosis and post × fibrosis), with a random intercept for each participant.

For planned sensitivity analyses included in the supplement, treatment-specific FVC % predicted trajectories were visualized using locally weighted scatterplot smoothing. This nonparametric method was selected instead of the linear spline modeling approach used for the primary analysis because of smaller sample sizes in each treatment arm and the limited interpretability of pretreatment trends in these patients. Locally weighted scatterplot smoothing allowed for flexible modeling of nonlinear patterns in treatment response without assuming a specific functional form. Each treatment group was plotted independently, with time 0 defined as the date of treatment initiation (ie, the date of the pretreatment FVC measurement), to illustrate changes in lung function after therapy (e-Appendix 1).

Descriptive statistics were used to summarize baseline demographic, clinical, radiographic, and treatment characteristics of the study cohort. Continuous variables were reported as mean (SD) or median (interquartile range [IQR]), as appropriate, and categorical variables were summarized as counts with corresponding percentages. Antigen exposure, radiographic features, and immunomodulatory therapy patterns also were described to contextualize heterogeneity within the cohort at the time of treatment initiation. All statistical tests were conducted with an α level of .05. Statistical analyses were conducted with R version 4.5.2 software (R Foundation for Statistical Computing).

## Results

### Participant Characteristics

Baseline characteristics of the 108 participants who met inclusion criteria for this analysis are outlined in Table 1. Overall, the average baseline FVC % predicted and Dlco % predicted were low in this cohort, indicating advanced disease before initiation of treatment. Most patients included in this study were treated with mycophenolate mofetil (63%), and prednisone was the second most common primary treatment (23.1%). Twenty patients were also started on prednisone at the time of initiation of additional immunomodulatory therapy. Eighty-six patients were included from the University of Kansas ILD Center and 22 patients from the University of Calgary. ILD clinicians identified antigens in 80 of the patients in this cohort who subsequently were treated with immunomodulation. Most patients had a visual fibrosis score of ≥ 10% (60.2%); however, usual interstitial pneumonia prevalence was low in this cohort (4.6%).

**Table 1:** Patient Characteristics (N = 108)

Variable	Data
Baseline characteristics	
Mean age at treatment initiation, y	68.2 (12.3)
Male sex	47 (43.5%)
Ever smoking	62 (57.4%)
Baseline FVC % predicted	64.9 (45.1-84.7)
Baseline Dlco % predicted	49.5 (32.8-66.2)
Antigen identified	80 (74.1%)
Radiographic features	
Usual interstitial pneumonia	5 (4.6%)
Visual fibrosis score ≥ 10%	65 (60.2%)
Immunomodulatory therapy	
Mycophenolate mofetil	68 (63%)
Combined prednisone and mycophenolate mofetil	18/68
Azathioprine	13 (12%)
Combined prednisone and azathioprine	2/13
Rituximab	2 (1.9%)
Prednisone	25 (23.1%)
Median follow-up period after treatment, y	1.86 (0.89-3.3)

Data are presented as No. (%), No./Total No., mean (SD), or median (interquartile range). Dlco = diffusion capacity of the lungs for carbon monoxide.

### Impact of Immunomodulation on Lung Function Trajectory: Overall Cohort

**Figure 1:**
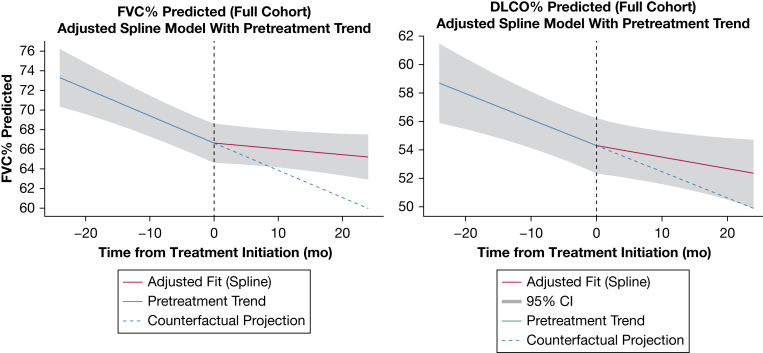
A, Graph showing adjusted FVC analysis of pretreatment FVC trend with counterfactual projection compared with measured FVC trend after initiation of immunomodulation showing +2.63% (*P* < .01; 95% CI, 0.72-4.54) when compared with pretreatment trend. B, Graph showing adjusted Dlco analysis of pretreatment Dlco trend with counterfactual projection compared with measured Dlco trend after initiation of immunomodulation showing +1.22% (*P* = .20; 95% CI, –0.64 to 3.08). Dlco = diffusion capacity of the lungs for carbon monoxide.

Five hundred ninety-seven FVC and 429 Dlco data points contributed to the primary outcomes. In the adjusted linear spline model, initiation of immunomodulation in this cohort was associated with a mean FVC % predicted improvement of +2.63% at 12 months (*P* < .01; 95% CI, 0.72-4.54]) when compared with the pretreatment trajectory. Initiation of immunomodulation was associated with a mean Dlco % predicted improvement of +1.22% at 12 months (*P* = .20; 95% CI, –0.64 to 3.08) (Fig 1).

### Impact of Visual Fibrosis Extent on Lung Function Trajectory Response to Immunomodulation

**Figure 2:**
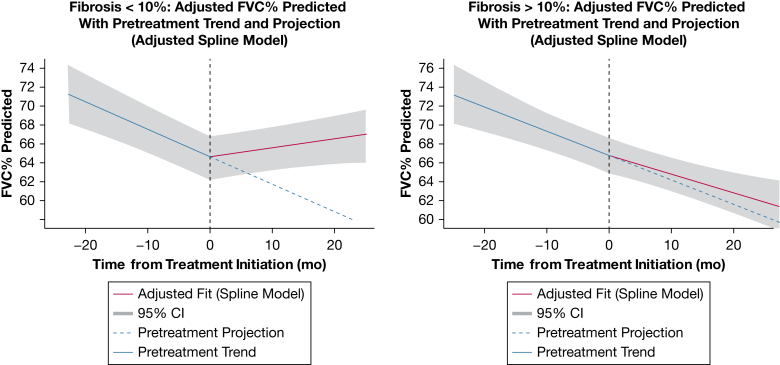
A, Graph showing adjusted FVC % predicted spline model with pretreatment trend and projection compared with measured adjusted fit in patients with visual fibrosis extent of < 10%. B, Graph showing adjusted FVC % predicted spline model with pretreatment trend and projection compared with measured adjusted fit in patients with visual fibrosis extent of > 10%.

**Figure 3:**
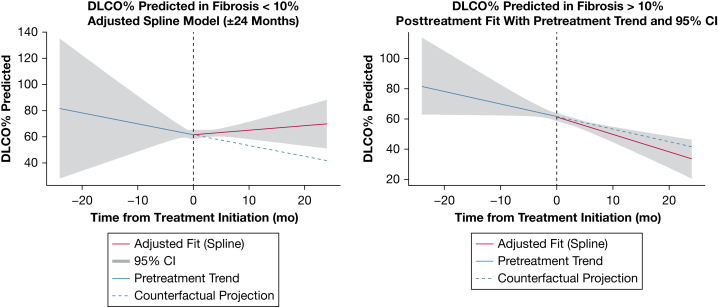
A, Graph of Dlco % predicted spline model showing comparison between pretreatment trend and counterfactual projection compared with the measured adjusted fit in patients with fibrosis extent of < 10%. B, Graph of Dlco % predicted spline model showing comparison between pretreatment trend and counterfactual projection compared with the measured adjusted fit in patients with fibrosis extent of > 10%. Dlco = diffusion capacity of the lungs for carbon monoxide.

No baseline differences in collected clinical and demographic features were noted across the 2 visual fibrosis extent groups (see e-Table 1 for characteristics of cohort based on visual fibrosis extent). In the group of patients with visual fibrosis extent < 10%, immunomodulation was associated with an improvement in FVC % predicted when compared with the pretreatment trajectory at 12 months (+5.83%; *P* < .01; 95% CI, 2.9-8.7) (Fig 2A). However, in the group of patients with visual fibrosis extent of ≥ 10%, the posttreatment trend after immunomodulation trended toward improvement in FVC % predicted when compared with the pretreatment trajectory at 12 months (+0.81%; *P* = .42; 95% CI, –1.2 to 2.8) (Fig 2B) was not significant. In the group of patients with lower visual fibrosis extent (< 10%), immunomodulation was associated with an improvement in Dlco % predicted when compared with the pretreatment trajectory (+13.9%; *P* < .01; 95% CI, 5.8-22.1) at 12 months (Fig 3A). Interestingly, in the group of patients with greater visual fibrosis extent (≥ 10%), the postimmunomodulation Dlco % predicted trend was worse than the pretreatment trend; however, this did not reach significance when compared with the pretreatment trajectory at 12 months (–4.3%; *P* = .17; 95% CI, –10.3 to 1.8) (Fig 3B). When modeled jointly using a single mixed-effects spline framework, posttreatment FVC trajectories differed significantly by fibrosis extent (*P* < .01), consistent with a slower rate of improvement among patients with ≥ 10% fibrosis (e-Fig 1).

### Individual Treatment Trends

See e-Figure 2 for postinitiation treatment trends based on individual treatments. Given the nonrandomized treatment selection in this cohort, no statistical tests were applied to these comparisons aside from visual trajectories.

## Discussion

This is the first empiric data, to our knowledge, to support the use of radiographic features to identify patients with HP most likely to respond to a specific treatment strategy. Although previously published research has identified the important role of genetic and genomic factors such as leukocyte telomere length, on clinical response to mycophenolate therapy,^21^ it remains difficult to operationalize these observations into clinical practice given the cost and lack of universal availability of these assays.^22^ In contrast to telomere length, the data presented herein identify a universally available clinical marker (visual fibrosis extent > 10%) that distinguishes immunomodulatory treatment response phenotypes that could impact clinical practice readily without added cost or complexity to implement.

Further, previous work by our group has explored quantitative machine learning approaches to quantify extents of radiologic features to predict treatment response in autoimmune ILD^17^; however, these types of quantitative approaches face regulatory hurdles to implementation and have limited generalizability on clinical impact. In contrast, visual extent of fibrosis as shown in these data could be clinically applicable and readily applied across centers and regions and in various clinical practices.

In HP, a reasonable search for markers that can identify inflammatory phenotypes that are most likely to positively respond to immunomodulation long has been pursued. Certainly, more invasive approaches such as lung biopsy showing evidence of lymphocytic inflammation^23^ or bronchoalveolar lavage fluid lymphocytosis^1^ could be reliable indicators of this phenotype. However, the operational and patient-risk considerations of these diagnostic tests need to be weighed against the potential of a noninvasive method such as radiographic characteristics.

We presented data on overall immunomodulatory impact on pulmonary function trajectory in this cohort first to evaluate how this cohort compares with previously published cohorts with HP asking similar questions. For instance, a previous cohort of patients with chronic HP (n = 70) showed a nonsignificant improvement in FVC trajectory and a significant Dlco improvement after patients were started on mycophenolate mofetil,^9^ whereas larger subsequent observational studies have shown no significant pulmonary function testing results change with immunomodulation in full cohort analyses.^24^ Given the differences in sample size and methodology and the inherent limitations of real-world data comparisons, we present our findings not to replicate prior studies, but rather to establish a point of reference. Importantly, the general consistency of our full cohort findings with prior reports supports the validity of our subsequent subgroup analyses focused on radiographic fibrosis extent.

These data are limited by their observational nature. To limit confounding by indication, we have constrained our analysis to before and after approaches using these patients as their own controls and have attempted merely to describe associations between trajectories based on immunomodulation use. However, even using this approach, potential concerns exist over extrapolating these data to other populations because of the confounding involved in provider treatment decisions. For these reasons, we view these data as hypothesis generating and believe these findings advocate for much-needed pragmatic, randomized studies of diagnostic and treatment strategies in HP, similar to those we have advocated for in autoimmune ILD.^25^ Although these data support the hypothesis of differential treatment response (pulmonary function trajectory) in HP based on this clinically available marker, these data are not positioned to assess differential safety or patient quality of life, which should be a priority for our field.

## Interpretation

Our findings underscore the potential for visual radiographic fibrosis extent—a widely accessible, reproducible clinical marker—to guide treatment decisions in chronic HP. Although these results are hypothesis generating, they highlight a critical opportunity to move beyond 1-size-fits-all treatment approaches toward stratified care in HP. The observed heterogeneity in treatment response, based on fibrosis extent, supports the urgent need for prospective, randomized studies to evaluate diagnostic and therapeutic algorithms. Such studies should prioritize both clinical outcomes and patient-centered measures, including safety and quality of life, to inform real-world practice meaningfully.

## Funding/Support

S. M. M. was supported by the 10.13039/100000050National Heart, Lung, and Blood Institute [Grant RO1HL176772] and the 10.13039/100000057National Institute of General Medical Sciences [Grant P20GM130423], 10.13039/100000002National Institutes of Health; the American Lung Association Dalsemer Award for Interstitial Lung Disease Research, the 10.13039/100003284Pulmonary Fibrosis Foundation Scholars Award, and the Kansas City Pulmonary Fibrosis Foundation.

## Financial/Nonfinancial Disclosures

The authors have reported to *CHEST* the following: M. H. reports research grants to his institution from Scleroderma Research Foundation, Boehringer Ingelheim, Bristol Myers Squibb, Calluna Pharmaceuticals, Avalyn Pharmaceuticals, Daewoong Pharmaceuticals, Genentech, Novartis, Puretech, Pliant Pharmaceuticals, Cumberland Pharmaceuticals, CSL Behring, Vicore, Fibrogen, and Kinevant Pharmaceuticals; has received speaker honoraria from Boehringer Ingelheim; serves on advisory boards for Boehringer Ingelheim, Avalyn Pharmaceuticals, and Trevi Pharmaceuticals; and reports nonfinancial involvement with the Kansas City Foundation for Pulmonary Fibrosis. K. A. J. reports institutional support from the University Hospital Foundation, Three Lakes Foundation, Lung Health Foundation, and the Canadian Institute for Health Research; reports personal consulting income, speaker honoraria, and travel support from Boehringer Ingelheim and honoraria from AbbVie; and serves on the PFOX trial Data Safety Monitoring Board. S. M. M. serves on an advisory board for Boehringer Ingelheim, Inc. None declared (C. N. M., B. M., A. M., E. I., S. P., G. L., M. C.).

## Supplementary Data

e-Online Data
