# Supplementary material for: Role of Radiographic Fibrosis Extent in Identifying Immunomodulatory Treatment Response in Hypersensitivity Pneumonitis
Source: Chest. 2025 Dec 18;169(6):1580–7. doi: 10.1016/j.chest.2025.12.007 (PMC13269666; doi:10.1016/j.chest.2025.12.007)

Supplementary table 1 comparing groups based on visual fibrosis extent of >/= 10% or less.

| **Variable** | **>/= 10% Fibrosis** | **<10% Fibrosis** | **P-Value** |
| --- | --- | --- | --- |
| Mean age at treatment initiation (SD) | 69.0 (11.8) | 67.0 (13.0) | 0.42 |
| Male, n (%) | 31 (47.7%) | 16 (37.2%) | 0.37 |
| Ever smoker, n (%) | 38 (58.5%) | 24 (55.8%) | 0.79 |
| Baseline FVC % Predicted Mean (SD) | 63.8 (18.6) | 66.5 (21.7) | 0.43 |
| Baseline DLCO % Predicted Mean (SD) | 48.3 (16.7) | 51.3 (16.6) | 0.28 |
| Antigen identified, n (%) | 47 (72.3%) | 33 (76.7%) | 0.63 |
| Usual interstitial pneumonia (UIP), n (%) | 4 (6.2%) | 1 (2.3%) | 0.39 |
| Mycophenolate mofetil, n (%) | 41 (63.1%) | 27 (62.8%) | 0.91 |
| Azathioprine, n (%) | 8 (12.3%) | 5 (11.6%) | 0.91 |
| Rituximab, n (%) | 2 (3.1%) | 0 (0.0%) | 0.21 |
| Prednisone, n (%) | 14 (21.5%) | 11 (25.6%) | 0.67 |

**Supplement Figure 1.**

**
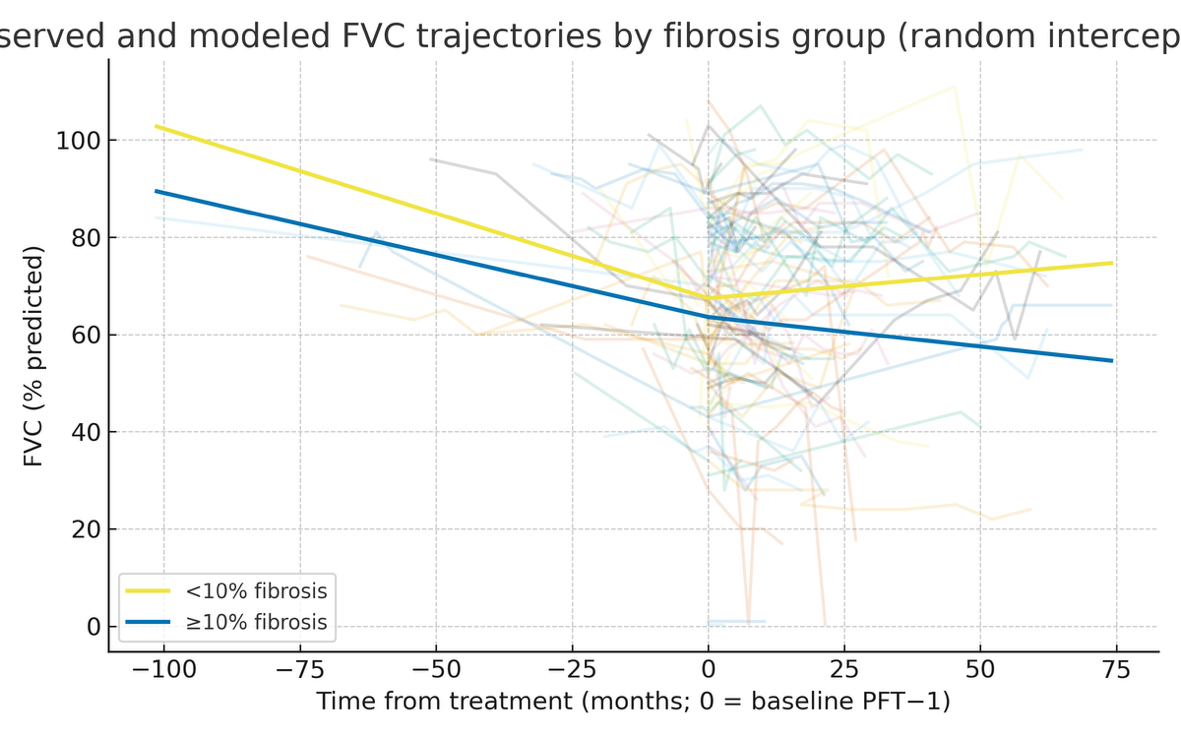
**

**Observed and modeled FVC trajectories by fibrosis extent.**
Spaghetti plots show individual patient trajectories for FVC (% predicted) before and after initiation of immunomodulatory therapy, stratified by visual fibrosis extent (<10 % (YELLOW) vs ≥10 % (BLUE)). Thick colored lines represent model-predicted mean trajectories derived from a linear mixed-effects spline model with a knot at treatment initiation (month 0) and random intercepts for each participant. Fixed effects included Time (pre-treatment slope), Post (post-treatment slope), Fibrosis extent, and their interaction terms. The model demonstrated a significant Post × Fibrosis interaction (p = 0.008), indicating that patients with ≥10 % fibrosis had a significantly slower post-treatment FVC trajectory compared with those with <10 % fibrosis.

Supplement Figure 2.

Specific post-treatment FVC trends by drug:


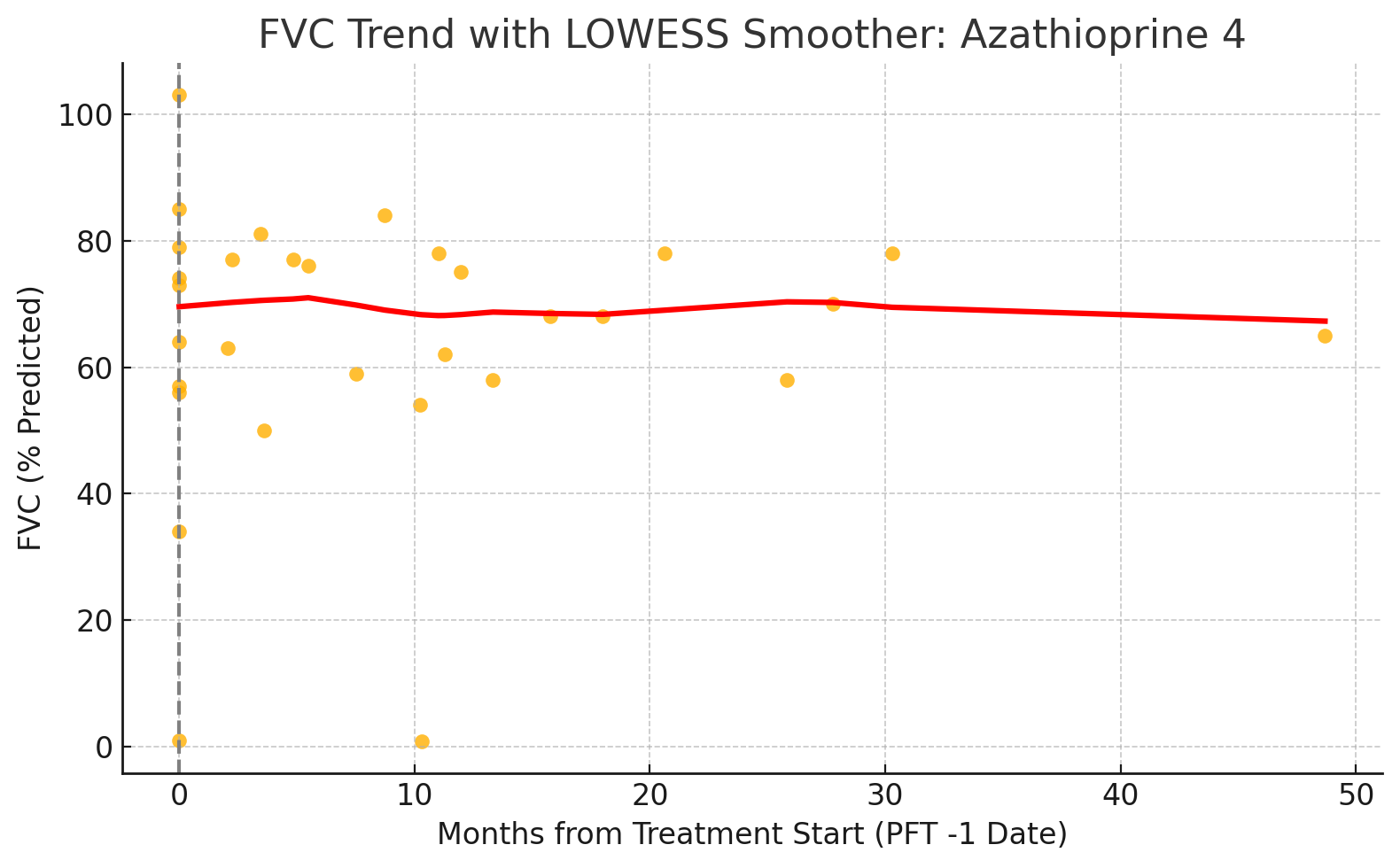


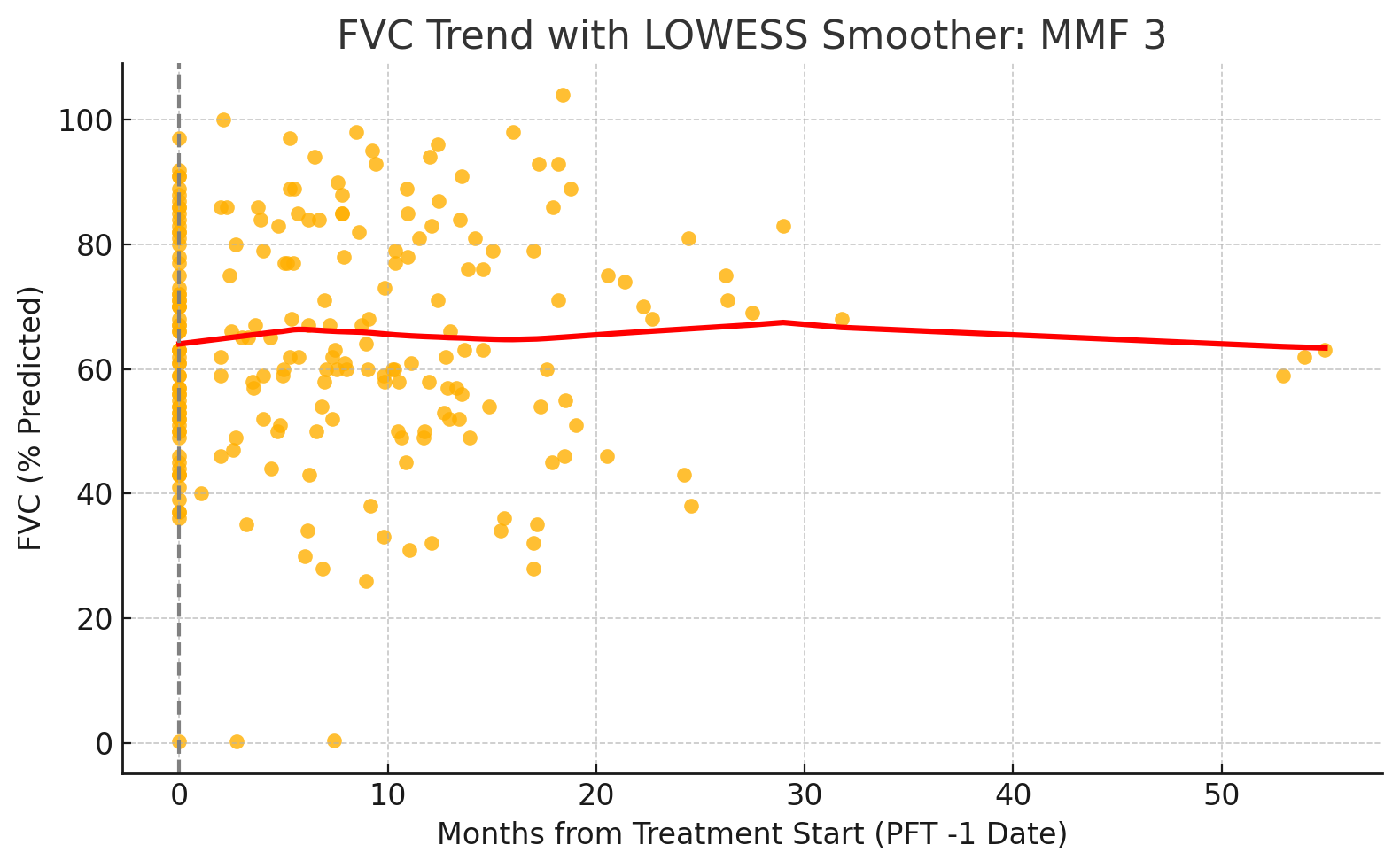


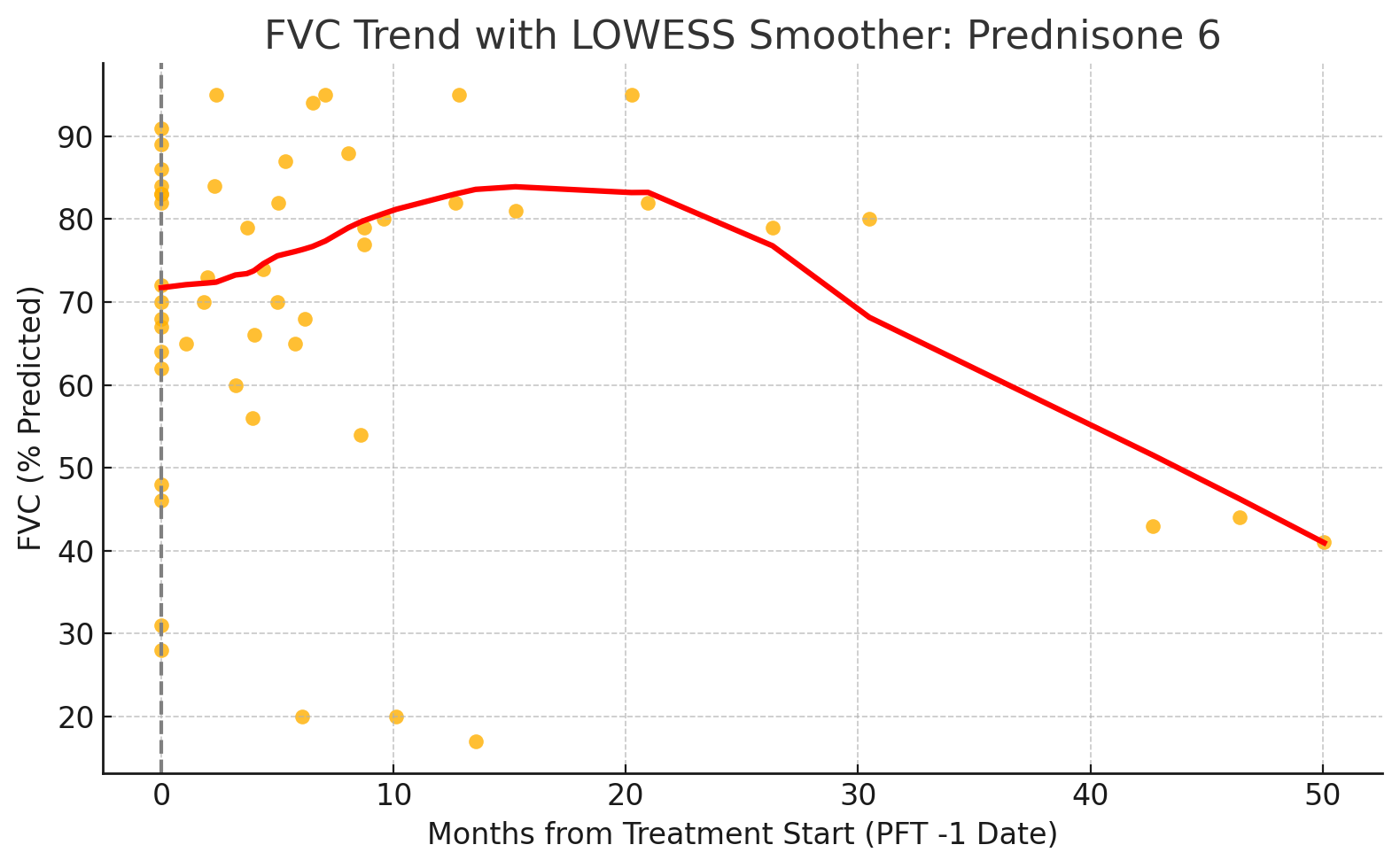


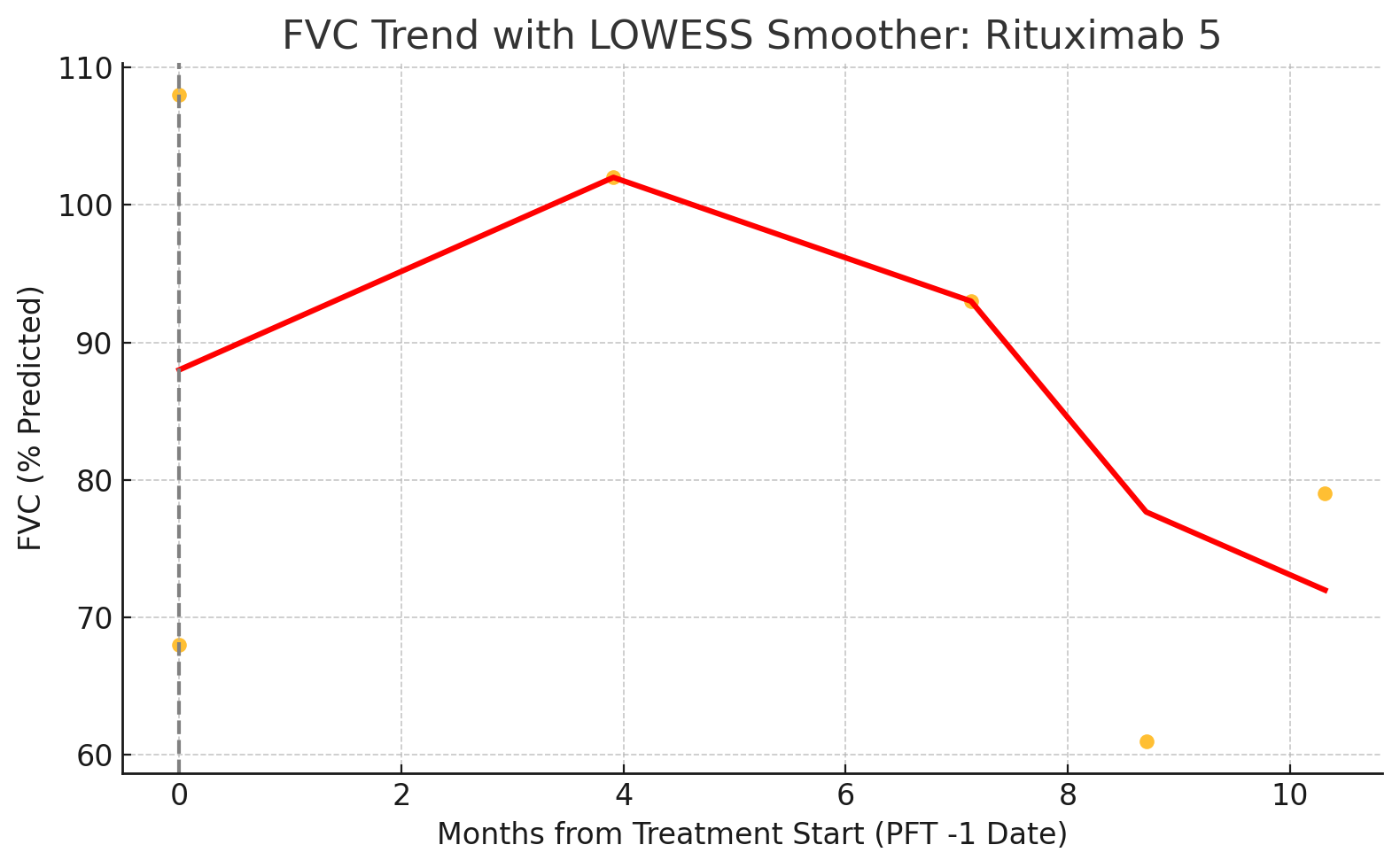

Supplement: e-Online Data [file mmc1.docx]
